# Begomoviral Movement Protein Effects in Human and Plant Cells: Towards New Potential Interaction Partners

**DOI:** 10.3390/v9110334

**Published:** 2017-11-09

**Authors:** Susanna Krapp, Christian Schuy, Eva Greiner, Irina Stephan, Barbara Alberter, Christina Funk, Manfred Marschall, Christina Wege, Susanne M. Bailer, Tatjana Kleinow, Björn Krenz

**Affiliations:** 1 Department Biologie, Lehrstuhl Biochemie, Universität Erlangen-Nürnberg, Staudtstr. 5, 91058 Erlangen, Germany; susanna.krapp@fau.de (S.K.); christian.schuy@fau.de (C.S.); eva.greiner@fau.de (E.G.); 2 Abteilung Molekularbiologie und Virologie der Pflanzen, Institut für Biomaterialien und Biomolekulare Systeme, Universität Stuttgart, Pfaffenwaldring 57, 70569 Stuttgart, Germany; irina.stephan@web.de (I.S.); barbara.alberter@web.de (B.A.); christina.wege@bio.uni-stuttgart.de (C.W.); tatjana.kleinow@bio.uni-stuttgart.de (T.K.); 3 Institute for Interfacial Engineering and Plasma Technology IGVP, Universität Stuttgart, Nobelstrasse 12, 70569 Stuttgart, Germany; christina.funk@igb.fraunhofer.de (C.F.); susanne.bailer@igb.fraunhofer.de (S.M.B.); 4 Institute for Clinical and Molecular Virology, Universität Erlangen-Nürnberg, 91054 Erlangen, Germany; manfred.marschall@viro.med.uni-erlangen.de; 5 Leibniz Institute DSMZ-German Collection of Microorganisms and Cell Cultures, Inhoffenstr. 7 B, 38124 Braunschweig, Germany

**Keywords:** *Geminiviridae*, begomovirus, Abutilon mosaic virus, Cleome leaf crumple virus, microtubule, movement protein, mammalian cells, plant cells, ectopic expression, tropism, cytoskeleton

## Abstract

Geminiviral single-stranded circular DNA genomes replicate in nuclei so that the progeny DNA has to cross both the nuclear envelope and the plasmodesmata for systemic spread within plant tissues. For intra- and intercellular transport, two proteins are required: a nuclear shuttle protein (NSP) and a movement protein (MP). New characteristics of ectopically produced Abutilon mosaic virus (AbMV) MP (MP^AbMV^), either authentically expressed or fused to a yellow fluorescent protein or epitope tags, respectively, were determined by localization studies in mammalian cell lines in comparison to plant cells. Wild-type MP^AbMV^ and the distinct MP^AbMV^: reporter protein fusions appeared as curled threads throughout mammalian cells. Co-staining with cytoskeleton markers for actin, intermediate filaments, or microtubules identified these threads as re-organized microtubules. These were, however, not stabilized by the viral MP, as demonstrated by nocodazole treatment. The MP of a related bipartite New World begomovirus, Cleome leaf crumple virus (ClLCrV), resulted in the same intensified microtubule bundling, whereas that of a nanovirus did not. The C-terminal section of MP^AbMV^, i.e., the protein’s oligomerization domain, was dispensable for the effect. However, MP expression in plant cells did not affect the microtubules network. Since plant epidermal cells are quiescent whilst mammalian cells are proliferating, the replication-associated protein Rep^AbMV^ protein was then co-expressed with MP^AbMV^ to induce cell progression into S-phase, thereby inducing distinct microtubule bundling without MP recruitment to the newly formed threads. Co-immunoprecipitation of MP^AbMV^ in the presence of Rep^AbMV^, followed by mass spectrometry identified potential novel MP^AbMV^-host interaction partners: the peptidyl-prolyl cis-trans isomerase NIMA-interacting 4 (Pin4) and stomatal cytokinesis defective 2 (SCD2) proteins. Possible roles of these putative interaction partners in the begomoviral life cycle and cytoskeletal association modes are discussed.

## 1. Introduction

Circular single-stranded DNA (ssDNA) viruses are the smallest viruses known to infect eukaryotes. The ssDNA viruses of the family *Geminiviridae* belong to the most devastating plant viruses causing heavy losses on food and cash crops [1]. Their genomes consist of one (monopartite) or two (bipartite) circular ssDNA molecules, which are packaged separately in twinned icosahedral particles, hence their name [2]. The small genomes (2.5 to 3.0 kb in size) multiply in the nuclei of host cells by complementary strand replication, rolling circle replication, and recombination-dependent replication [3,4]. Due to its replication in nuclei, geminivirus DNA has to cross two distinct barriers for systemic spread: the nuclear envelope and the plasmodesmata. The majority of begomoviruses within the family *Geminiviridae* possess a bipartite genome designated DNA A and DNA B, where DNA B encodes two movement-associated proteins, named nuclear shuttle protein (NSP) and movement protein (MP) (reviewed in [4,5]). The MP of the begomovirus Abutilon mosaic virus (AbMV), a phloem-limited virus [6,7], might exploit the cellular membrane flow from the endoplasmic reticulum (ER) to the plasma membrane via plasmodesmata into the adjacent cell [8] or by stromules to facilitate intracellular movement [9,10,11]. However, functional details of this process still remain elusive. Two models have been proposed for a cell-to-cell transport: the “couple-skating” model [8,12,13,14,15,16] and, alternatively, the “relay race” model [17,18,19,20]. To shed more light onto the mechanisms of geminivirus trafficking, new experimental model systems may be helpful, in combination with strategies to identify host-encoded interaction partners.

Only three interacting host factors have been identified for MPs of bipartite begomoviruses so far: Synaptotagmin A [21,22,23], a heat shock cognate 70 kDa protein (cpHSC70-1) [10] and histone H3 [24]. Lewis and Lazarowitz have used the yeast son of sevenless (SOS) recruitment screen to identify *Arabidopsis* proteins that interacted with MP of cabbage leaf curl virus (CaLCuV). They used an MP missense mutant with two alanine substitutions at positions 112 and 113, which, unlike intact MP, did not localize to or near the plasma membrane in plant or insect cells [22]. Krenz and colleagues [10] employed a truncated version of MP^AbMV^ in a yeast-two-hybrid assay to identify cpHSC70-1 as the interaction partner. Zhou et al. [24] used a biochemical approach to identify host factors interacting with the NSP and MP of the geminivirus bean dwarf mosaic virus (BDMV). In these studies, the host nucleoprotein histone H3 was found to interact with both the NSP and MP [24]. To date, no other host interaction partner has been identified for begomoviral MPs.

The limited knowledge of the transport complex/cytoskeleton interplay during geminivirus infection in planta suggested the initial functional evaluation of viral proteins in a well-characterized heterologous system, for which mammalian cells were chosen for this work. Numerous studies have revealed that animal viruses depend on cytoskeleton components for intracellular movement [25]. The three types of cytosolic filaments, i.e., actin filaments, intermediate filaments (IF), and microtubules (MT) form an organized network structure with direct links [26]. Animal viruses were shown to hijack motor proteins of MT and the F-actin network to transport viral components through the host cell [27]. In plants, several investigations of MPs from different viruses revealed interaction with the endoplasmic reticulum (ER), as established for the tobamovirus tobacco mosaic virus (TMV) MP [28], the begomovirus tomato yellow leaf curl virus V1 [29], BDMV MP [29], and squash leaf curl virus (SLCV) MP [30], suggesting that MP–ER interactions may be important for intracellular trafficking. Targeting of the plasma membrane is possibly mediated via the microtubule- and actin-dependent control of the plant endomembrane system [31]. For example, distinct MT-ER junctions, to which MP^TMV^ localizes, may anchor the ER-actin network to the plasma membrane and provide an interlinkage for an intracellular transport pathway. Further accumulation of MPs from the begomovirus CaLCuV, the potyvirus turnip mosaic virus, the tobamoviruses TMV, and tobacco vein clearing virus at plasmodesmata, indicate subsequent intercellular transfer occurs most likely by an endocytotic recapture pathway dependent on the synaptotagmin-regulated route [23]. A number of viruses also displayed interactions with MT and/or actin filaments in plants, which are most likely involved in several aspects of viral pathogenesis, such as the cytoskeleton-mediated formation of cytoplasmic viral replication complexes (VRC) and cell-to-cell transport [32,33,34,35]. Interactions with MTs were similarly shown for MP^TMV^ in mammalian cells, also leading to a disruption of MT organization and centrosome function in these cells [36]. In mammalian cells, MTs play a role in concentration and aggregation of proteins at microtubule-organizing centers (MTOCs) and, thus, in later degradation of these aggreosomes by proteasomes and/or autophagy [37]. The association of plant RNA virus MPs with MTs has led to the assumption that they exploit aggreosomal mechanisms for VRC formation and a controlled balance of their protein concentrations including MPs [31], and that MTs are implicated in the transport of mobile VRCs and viral genomes. An association of geminiviral MPs with cytoskeleton components has not been identified so far.

In this study, the MPs of the begomoviruses AbMV, Cleome leaf crumple virus (ClLCrV), the nanovirus pea necrotic yellow dwarf virus (PNYDV), and the transmission component P2 of the caulimovirus cauliflower mosaic virus (CaMV) were investigated. Viral proteins fused to enhanced yellow fluorescent protein (EYFP) were expressed in human epithelial cervix carcinoma cells (HeLa) and COS-7 (African green monkey kidney cells) to investigate the subcellular localization via microscopy. By use of these heterologous cells, a relationship between begomovirus infection and the MT network has been demonstrated for the first time. To detect potential similar interactions between MT and a begomovirus MP in host cells, the viral protein was ectopically expressed in plants. In the presence of co-expressed viral replication-associated protein (Rep) that is known to mediate cell cycle progression, MP effects on the MT network were identified as a new characteristic of this virus infection. They, however, differed from the MP’s impact on animal cells, as shown in the following, which led to the identification of potential new MP plant interaction partners.

## 2. Materials and Methods

### 2.1. Cell Culture, Plants, and General Methods

The permanent cell lines HeLa (human epithelial) and COS-7 (African green monkey kidney cells), verified to be free of mycoplasma, were cultured in Dulbecco’s modified Eagle’s medium (DMEM; Life Technologies/Gibco, Carlsbad, CA, USA) containing 10% heat-inactivated fetal bovine serum at 37 °C in an air-5% CO_2_ atmosphere at constant humidity. *N. benthamiana* plants were grown in an insect-free S1 greenhouse with 16 h light 25 °C/8 h darkness 22 °C conditions.

### 2.2. Construction of Expression Plasmids for Cultured Cells

Genes of MP^ClLCrV^ (FN436000), MP^PNYDV^ (NC_023156), MP^AbMV^ (NC_001929), P2^CaMV^ (kindly provided by Martin Drucker, INRA, Montepellier, France), and *Homo sapiens* microtubule-associated protein 4 (MAP4, kindly provided by Benedikt Kost, University Erlangen-Nuremberg) were amplified by PCR using the primers listed in Table S1. The open reading frames (ORFs) of the viral proteins were ligated into pEYFP-N1 (Clontech, Mountain View, CA, USA) via the *EcoR*I-*Bam*HI sites, and that of MAP4 was ligated into pDsRed2-N1 via the *Hind*III-*Eco*RI sites. The human influenza hemagglutinin (HA) -tagged MP^AbMV^ gene was inserted into the pEYFP-N1 sequence at the *Eco*RI-*Not*I restriction sites and, therefore, the EYFP coding sequence was removed. The sequences encoding the different domains of MP^AbMV^ were produced by PCR using the primer pairs indicated in the Table S1. Fragments were digested with *Eco*RI-*Bam*HI, and inserted into the corresponding sites of pEYFP-N1. The mutant MP^AbMV^_K112A/D113A_ was generated by overlap PCR and, as before, cloning into pEYFP-N1 via *Eco*RI-*Bam*HI sites. The encoded MP differs from the wild-type protein by a Lys-to-Ala substitution at amino acid position 112 and an Asp-to-Ala substitution at amino acid position 113 [38]. In addition, a Gateway entry clone carrying the MP-coding region inclusive stop codon was generated by *Bam*HI-*Xho*I release of the respective fragment from a pGEM-T construct [14] and insertion of it into the *Bam*HI-*Xho*I sites of pENTR11 (Invitrogen, Carlsbad, CA, USA). The analogous entry construct of the *MP* gene omitting the stop codon (pDONR207-BC1) was described previously [10]. The MP fragments were recombined into Gateway destination vector versions of pCR3 (Invitrogen) for expression in mammalian cells (pCR3-*N*-myc for N-terminal fusion to a Myc epitope and pCR3-*C*-myc for C-terminal fusion to a Myc epitope).

### 2.3. Construction of Expression Plasmids for Plants

MP^AbMV^, MP^AbMV^_aa1-180_, *Arabidopsis thaliana* (A.t.) Pin4 (AT1G26550), and *Solanum lycopersicum* (S.l.) SCD2 (XP_004235837) fragments were PCR amplified, first inserted into the vector pENTR-D/TOPO (Invitrogen), sequenced, and then transferred into vectors pK7FWG2.0 or pK7RWG2 [33] with L/R-Clonase^TM^ II enzyme mix (Invitrogen). Gateway^®^ compatible vectors pRB-*C*-VenusN173 and pRB-*C*-VenusC155 [39] were used for bimolecular fluorescence complementation (BiFC) experiments. MP^AbMV^, MP^AbMV^_aa1-180_, A.t.Pin4, and S.l.SCD2 pENTR-D/TOPO clones were recombined into these BiFC destination vectors as above. MAP4 and Rep^AbMV^ genes were amplified by PCR and MAP4 was ligated into pSK35S:mCherry at the *EcoR*I-*Xba*I sites, and Rep^AbMV^ was ligated into pSK35S:mtagBFP at the *Spe*I-*Bam*HI sites. The specific domain sequences of MP^AbMV^ were amplified by PCR, digested with *Eco*RI-*Bam*HI, and inserted into the corresponding sites of pSK35S:CVN and pSK35S:CVC, respectively. The primers utilized for the amplification are listed in Table S2. All constructs were confirmed by sequencing.

### 2.4. Transfection of Cultured Cell Lines and Immunofluorescence Analysis

For the transfection of cells, ~5 × 10^4^ adherent cells were seeded onto 12-mm round glass coverslips in a 24-well plate filled with 1 mL growth medium per well, 24 h prior to transfection. Then, 1 µg of plasmid DNA was diluted in 100 µL of serum-free DMEM. 2 µL transfection reagent (TurboFect, Thermo Scientific, Waltham, MA, USA) was vortexed and added to the diluted DNA, mixed, and incubated for 20 min at room temperature. The transfection reagent/DNA mixture was pipetted drop-wise to each well, and the plate was rocked gently immediately after adding the mixture. The cells were incubated for 48 h at 37 °C in an air-5% CO_2_ incubator and then fixed for immunofluorescence analysis in phosphate-buffered saline (PBS), pH 7.4, containing 4% formaldehyde (freshly prepared from paraformaldehyde) for 20 min. After being washed in PBS for 10 min, cells were permeabilized in PBS, pH 7.4, containing 0.3% Triton X-100 for 1 min. In cases where cells were stained with a β-tubulin antibody, cells were fixed with ice-cold methanol at −20 °C for 10 min and permeabilized as above. The cells were washed twice in PBS for 10 min each before blocking in PBS, pH 7.4, supplemented with 5% goat serum and 0.3% Triton X-100 for 45 min before application of the antibody. The preparations were stained for 2 h with rhodamine phalloidin (Thermo Fisher, Osterode, Germany) for visualization of actin filaments. MP^AbMV^:HA was detected using either a primary anti-HA immunoglobulin G (IgG) mouse antibody MMS-101P (Covance Inc., Princeton, NJ, USA) diluted 1:500, or a primary anti-HA IgG rat antibody clone 3F10 (Roche Diagnostics GmbH, Mannheim, Germany) diluted 1:100. Primary antibodies detecting host proteins were directed against microtubules (anti-β-tubulin IgG mouse antibody T4026, Sigma-Aldrich, St. Louis, MO, USA, diluted 1:400); intermediate filaments (anti-α-vimentin IgG-mouse antibody V6630, Sigma-Aldrich, 1:250); or integral ER protein calnexin (anti-calnexin IgG mouse antibody C7617, Sigma-Aldrich, 1:200). All primary rat IgG antibodies were detected using secondary Cy5-conjugated goat anti-IgG rat antibody 112-175-167 (Jackson Immuno Research, West Grove, PA, USA) diluted 1:64. All primary mouse IgG antibodies were detected using secondary fluorescein isothiocyanate (FITC)-coupled sheep anti-IgG mouse antibody F3008 (Sigma-Aldrich) diluted 1:64. Primary antibodies were applied to cells at room temperature for 2 h; the secondary antibodies were applied at room temperature for 1 h in the dark. After incubation with a primary and secondary antibody, cells were washed three times for 5 min each with PBS. Cells were rinsed with distilled water and mounted in Vectashield (Vector Laboratories) supplemented with 50% glycerol and 2.5% 1,4-diazobicyclo[2.2.2]octane (DABCO, Sigma-Aldrich). Fluorescence microscopy was performed with a confocal laser scanning microscope (Leica TCS SP5 II, Optical imaging center Erlangen, Erlangen, Germany).

Alternatively, indirect immunofluorescence analysis of transfected cells, presented in Figure S1, was essentially done as described by Striebinger and colleagues [40]. Plasmid transfection was performed by using the Effectene Transfection Reagent Kit according to manufacturers’ recommendations (Qiagen, Hilden, Germany). Twenty h post-transfection, HeLa cells were subjected to immunofluorescence analysis. Immunodetection of Myc-tagged MP^AbMV^ used a monoclonal anti-Myc antibody IgG1 from mouse (undiluted cell culture supernatant provided by Jens von Einem, Universität Ulm) and a secondary anti mouse antibody from goat tagged with Alexa Fluor 555 (diluted 1:500; Invitrogen). Unmodified MP^AbMV^ was detected using an anti-MP polyclonal rabbit antiserum (diluted 1:500; [38]) and a secondary goat anti-rabbit antibody either coupled to Alexa Fluor 488, or Alexa Fluor 555 (diluted 1:500; all Invitrogen). Fluorescence signals within cells obtained through indirect immunolabelling were monitored by confocal microscopy in a different setup (LSM 710, Zeiss, shown in Figure S1 only), and images were processed by Adobe Photoshop (Adobe Photoshop Elements 10.0, Adobe Systems San Jose, California, USA) or Zen Lite software (Zen Lite software Version 2.3, Zeiss, Jena, Germany).

### 2.5. Agro-Infiltration Assay and Microscopy

Agro-infiltration assay and microscopy with the confocal laser scanning microscope (CLSM) Leica SP5 II were performed as described in [41].

### 2.6. Immune-Affinity Capture of Proteins, Off-Bead Tryptic Digest, Nano-liquid chromatography (LC), and Tandem Mass Spectrometry (MS/MS) Analysis

Immune-affinity capture of proteins with RFP-trap^®^_M was performed according to the manufacturer ChromoTek (Munich, Germany) and the samples further processed as described in [41].

### 2.7. Biolistic Inoculation of Plant Tissue

Biolistic inoculation of plant tissue was performed according to Zhang et al. [8].

### 2.8. Tobacco Rattle Virus (TRV)-Based Virus-Induced Gene Silencing (VIGS) in N. benthamiana

ORF of Pin4 from *N. benthamiana* was amplified by RT-PCR, inserted into pTRV2 via the *Bam*HI restriction site and named pTRV2-NbPin4. For VIGS assays, pTRV1 or pTRV2 and its derivative pTRV2-NbPin4 were introduced into the *Agrobacterium* strain C58C1. *Agrobacterium* cultures at OD_600_ = 0.1 containing TRV or TRV-derivative plasmids were mixed in 1:1 ratio and infiltrated in leaves of 5-leaf stage plants using a 1-mL needleless syringe. For experiments in which the suppression effect of NbPin4 on AbMV was investigated, these plants received a secondary inoculation with pBK-TR224 [12] by biolistic bombardment. Plants were monitored weekly.

### 2.9. Quantitative PCR (qPCR) Analysis

Virus DNA was extracted from plant tissue as described in Noris and Miozzi [42]. The quality was confirmed by gel electrophoresis. qPCR was performed on 1 mL diluted (1:5) DNA in 20 µL reactions using Brilliant II SYBR^®^ Green QPCR Master Mix (Agilent, Waldbronn, Germany) in an Agilent Technologies AriaMX Real Time PCR System (Oberhaching, Germany). Gene-specific primers amplify a fragment within the AC1 ORF (qPCRAbMVRepfor: 5′-TTCCCTGTCCTTGAATCACC-3′, qPCRAbMVReprev: 5′-GTGGGCGGATGATTATTTTG-3′). Relative quantification of the AbMV DNA A content adjusted for efficiency was performed using PCR Miner [43] with primer pair: 25S-rRNA-F: 5′-ATAACCGCATCAGGTCTCCA-3′ and 25S-rRNA-R: 5′-CCGAAGTTACGGATCCATTT-3′. Reference genes were only used if their stability values were within advised limits (M < 0.5 and C*_t_* < 0.25) [44].

## 3. Results

### 3.1. Expression of MP^AbMV^ in Mammalian Cell Lines Induced Filamentous Structures

**Figure 1:**
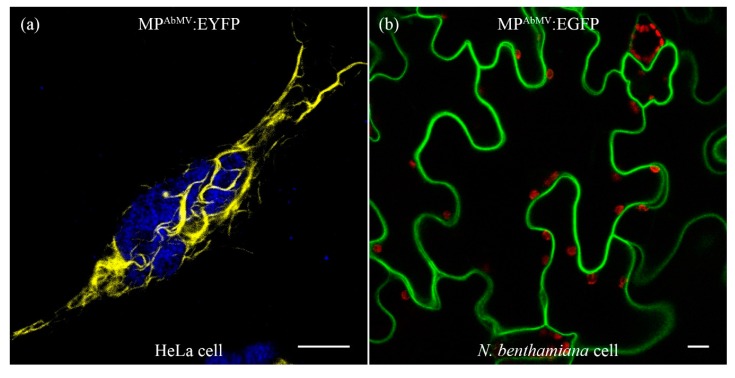
Localization of Abutilon mosaic virus (AbMV) MP (MP^AbMV)^ either fused to EYFP (**a**, MP^AbMV^:EYFP, yellow signal) in HeLa cells, or fused to EGFP (**b**, green signal) in epidermal cells of *N. benthamiana* plants. Blue signal in (**a**) represents 4′,6-diamidino-2-phenylindole (DAPI) staining; red signal in (**b**) represents chloroplast autofluorescence. Bar represents 10 µm.

**Figure 2:**
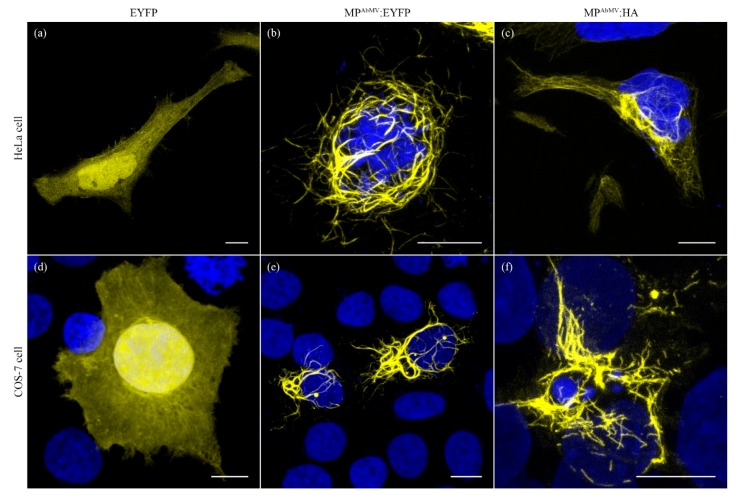
Cellular localization studies of EYFP, MP^AbMV^ and MP^AbMV^:HA (**a**–**c**) in HeLa and (**d**–**f**) COS-7. Blue signals represent DAPI staining (**b**–**f**). Bars represent 10 µm.

MP^AbMV^, with C-terminal fusion to enhanced yellow fluorescent protein (EYFP) (MP^AbMV^:EYFP), was expressed under the control of the cytomegalovirus (CMV) immediate early promoter in HeLa cells for investigating its subcellular localization by CLSM. MP^AbMV^:EYFP signals appeared as curled filamentous threads throughout the cell (Figure 1a), whereas freely expressed EYFP analyzed in parallel (after transfection of empty pEYFP vector) accumulated in the nucleus and cytoplasm (Figure 2a). This localization within a eukaryotic cell represented a new feature of MP^AbMV^. MP^AbMV^:enhanced green fluorescent protein (EGFP) expressed in *N. benthamiana* epidermal cells was observed to localize to the cell periphery (Figure 1b), presumably in the plasma membrane, but never in such filamentous threads.

To rule out that the prominent MP-dependent phenotype was restricted to this particular cell line, COS-7 cells were transfected with the same MP^AbMV^:EYFP construct or the empty pEYFP vector control, yielding essentially the same results. Hence the MP^AbMV^:EYFP fluorescence accumulated in filamentous structures in two distinct mammalian cell lines (Figure 2b,e), whereas free EYFP was dispersed (Figure 2a,d). To ensure that the EYFP portion was not responsible for the formation of the filamentous MP^AbMV^:EYFP structures, the EYFP-tag was substituted with an HA- or a Myc-tag, respectively. The expression of the MP^AbMV^:HA protein in both cell lines or of MP^AbMV^:Myc in HeLa cells and its detection by antibody treatment against the epitope revealed comparable threads distributed throughout the cells (Figure 2c,f and Figure S1). Finally and importantly, the fibrous phenotype could also be observed after in situ immunodetection of MP^AbMV^ expressed without any tag (Figure S1), which shows that the fusion of a fluorescent reporter protein to the MP’s C-terminus most likely did not influence its subcellular localization. A different result was observed when a tag was fused to the N-terminus of MP^AbMV^. Here, not only the thread phenotype could be observed, but in addition, aggregates close to the nuclei and a homogenous background distribution within the cytoplasm occurred (Figure S1).

### 3.2. Filamentous Structures Arise with Another Begomoviral MP as Well, but Not for an Unrelated Viral MP

To test whether other plant virus MPs accumulated in comparable filamentous threads inside mammalian cells, two other MPs were screened in the same system: the MP of a related virus in the family *Geminiviridae*, the bipartite New World begomovirus ClLCrV, and the MP of an ssDNA virus in the family *Nanoviridae*, the nanovirus PNYDV. Additionally, the aphid transmission component P2 of a double-stranded DNA (dsDNA) virus in the *Caulimoviridae* family, CaMV, was included in the experiments. All three proteins were expressed in HeLa cells in C-terminal fusion with EYFP. Both the nanovirus MP and the caulimovirus P2 did not present a filamentous phenotype as did the MP^AbMV^. Instead, the MP^PNYDV^:EYFP localization was comparable to the expression pattern of free EYFP. P2^CaMV^:EYFP signals appeared as inclusion bodies within the cells, as described for the P2 foci observed in protoplasts [45]. The MP^ClLCrV^:EYFP signal, however, revealed the same phenotype as the MP^AbMV^ in both animal cell lines (Figure S2).

### 3.3. MP^AbMV^ Induces Re-Organization of Microtubules and Intermediate Filaments

**Figure 3:**
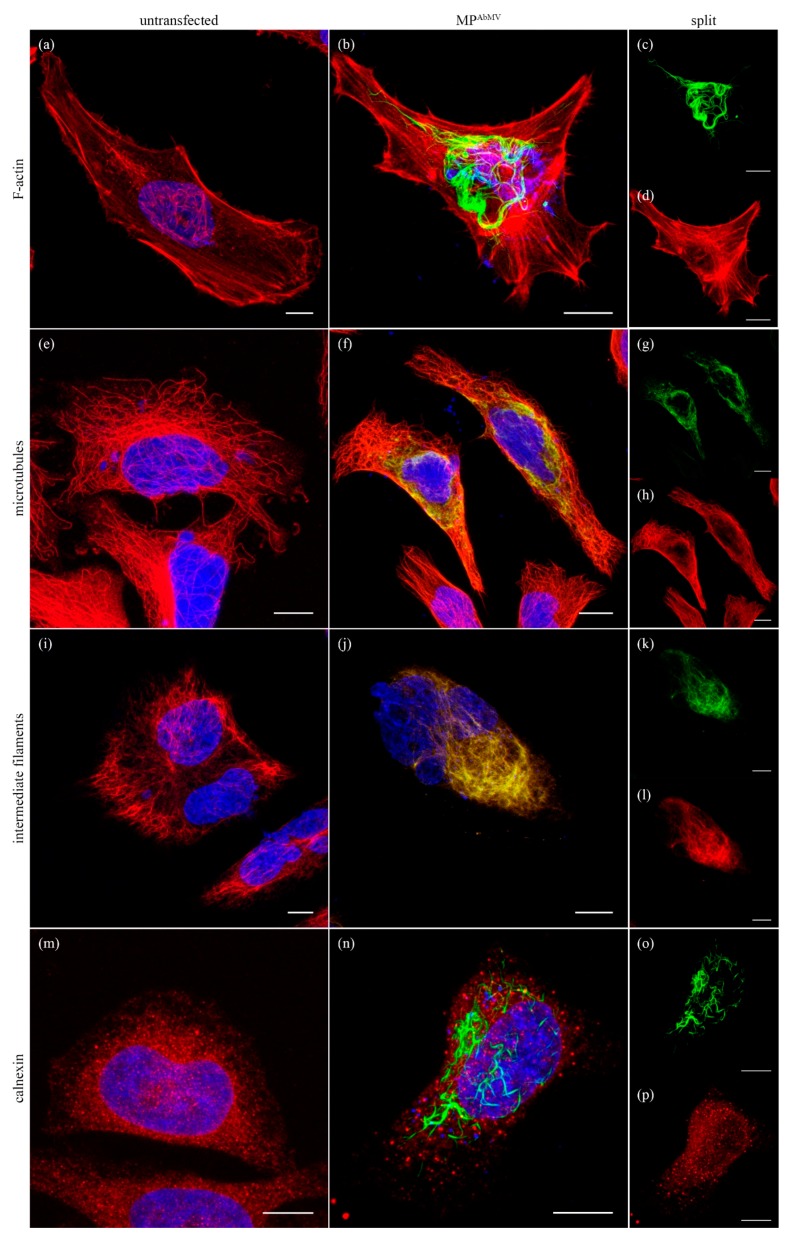
Analysis of the MP^AbMV^ influence on the cytoskeleton. (**a**) F-actin; (**e**) microtubules (MT); (**i**) intermediate filaments (IF), and (**m**) endoplasmic reticulum (ER) network were stained in the absence or presence (**b**,**f**,**j**,**n**) of MP^AbMV^; (**c**,**d**,**g**,**h**,**k**,**l**,**o**,**p**) show split channels, respectively. Cytoskeleton stainings are shown in red, MP^AbMV^ in green. Blue signals represent DAPI staining of DNA. Bar represents 10 µm.

The MP^AbMV^:EYFP accumulation patterns led to the assumption that MP^AbMV^ interacted with or re-organized a component of the cytoskeleton. Therefore, MP^AbMV^-expressing HeLa cells were fixed and either co-stained with rhodamine-phalloidin to visualize F-actin filaments (Figure 3a–d) or treated with antibodies against β-tubulin (Figure 3e–h), α-vimentin (Figure 3i–l), or calnexin (Figure 3m–p) for visualization of MT, intermediate filaments (IF), or endoplasmic reticulum (ER), respectively. The F-actin filaments and ER network were virtually unaltered upon MP accumulation (Figure 3b–d,n–p) and did not colocalize with MP^AbMV^. In contrast, MP^AbMV^ was detected at the MT cytoskeleton and the IF (Figure 3f–h,j–l), compared to the MP-untransfected control cells. The association of MP^AbMV^ to the MT appeared to induce bundling of the MT, since the control cells devoid of MP^AbMV^ exhibited a normal radial microtubule organization consisting of MT that extended from the centrosomal region close to the nucleus toward the cell periphery (Figure 3e), and evenly distributed IF (Figure 3i).

### 3.4. MP^AbMV^ Does Not Stabilize Microtubules

**Figure 4:**
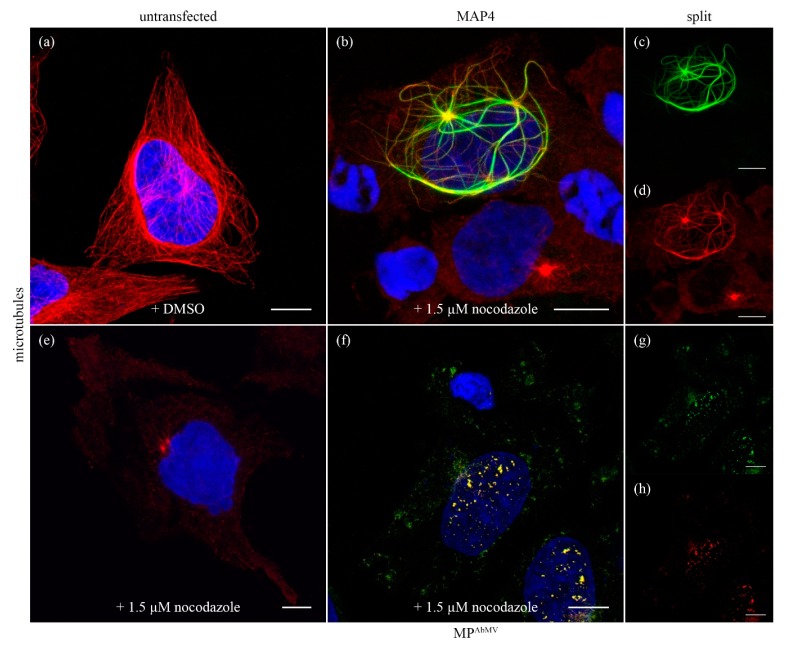
Microtubule stabilization assay with nocodazole. (**a**) MT staining in DMSO treated cell, (**b**) nocodazole treated cell expressing MAP4:EYFP where MT are stained in red. (**c**,**d**) show split channels of (**b**). (**e**) MT staining (red signal) in nocodazole treated cell, (**f**) nocodazole treated cell expressing MP^AbMV^:EYFP, again MT are stained in red. (**g**,**h**) show split channels of (**f**). Microtubuli staining is shown in red, microtubule-associated protein 4 (MAP4) or MP^AbMV^ in green. Blue signals represent DAPI staining of DNA. Bar represents 10 µm.

MP^AbMV^-expressing HeLa cells were treated with nocodazole, a drug interfering with the dynamic tubulin polymerization, to investigate whether MP^AbMV^ might not only act as a MT bundling agent, but potentially even as a microtubule-stabilizing protein. Under the conditions applied, nocodazole-treated cells displayed disruption of the microtubules network (Figure 4e) compared to the control cells (Figure 4a). Two remaining small dots brightly labelled by β-tubulin-specific immunostaining most likely represented the MTOCs. Expression of the microtubule-binding domain of the MAP4, fused to dsRed, stabilized the network against nocodazole treatment, as expected (Figure 4b–d). In contrast, MP^AbMV^ could not maintain the filamentous structures in nocodazole-treated cells, but accumulated with β-tubulin in aggregates (Figure 4f–h).

### 3.5. The MP^AbMV^ Oligomerization Domain Is Dispensable for Interaction with the Microtubule Filaments

**Figure 5:**
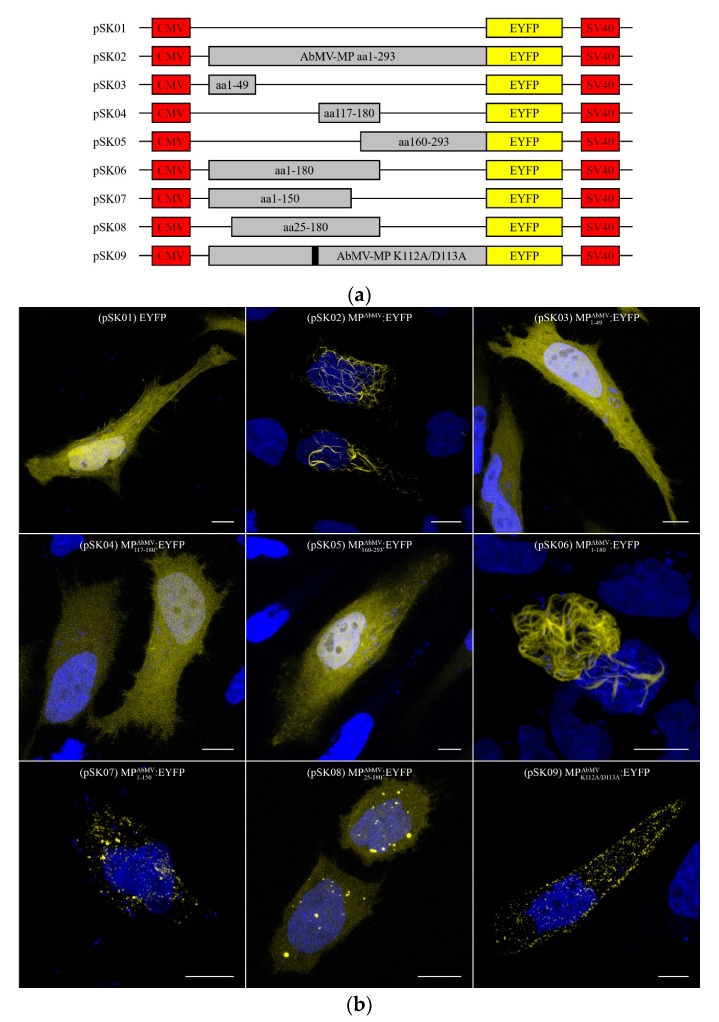
(**a**) Schematic representation of MP^AbMV^ deletion mutants; (**b**) Cellular localization studies of MP^AbMV^ deletion mutants. MP^AbMV^:EYFP signals in yellow, blue signals represent DAPI staining of nuclei. Bar represents 10 µm.

HeLa cells were then transiently transfected with a series of expression clones, encoding MP^AbMV^:EYFP deletion mutants, to determine which domains of MP^AbMV^ are necessary and sufficient for its capacity to maintain the localization phenotype of the wild-type protein. Figure 5a shows the domains of MP^AbMV^ and the set of constructs created to express MP^AbMV^:EYFP deletion mutants, which were chosen according to previously described domains in Zhang et al. [46], Frischmuth et al. [14], and Krenz et al. [10]. These are the MP pilot domains from amino acids (aa) 1–49 (pSK03), the anchor domain from aa 117–180 (pSK04), and the oligomerization domain from aa 160–293 (pSK05). After expression in the mammalian cells, all four mutants did neither colocalize with the MT network nor induce MT bundling, but behaved such as free EYFP (pSK01). An exception represented the fourth constructed domain from aa 1–180 (pSK06); this mutant exhibited the same phenotype as the wild-type MP^AbMV^:EYFP (pSK02) (Figure 5b). Hence, this putative domain is named “association domain” in the following. To narrow down the minimal “association domain”, mutant constructs were generated with truncated N- or C-termini of this domain. The cropped mutants of this part did not reveal the filamentous structure (pSK07 + pSK08), leading to the assumption that the minimal association domain spans from aa 1–180. In addition, an amino acid substitution mutant was created (pSK09) according to Sanderfoot and Lazarowitz [38]. The MP^AbMV^K112A/D113A:EYFP mutant showed punctuated aggregations at the cell periphery in HeLa cells (pSK09).

### 3.6. Expression of MP^AbMV^ with and without Its Oligomerization Domain Has No Impact on the Microtubules Network in N. benthamiana Plant Cells

To determine whether the expression of MP^AbMV^ exerted the same effect on the MT network in plant cells as in mammalian cells, MP^AbMV^ in C-terminal fusion to EGFP was expressed under the control of the CaMV 35S promoter in *N. benthamiana* plants, and its localization was examined by CLSM. The MAP4 domain fused to mCherry was used as a microtubule marker. The CLSM analysis revealed that neither MP^AbMV^:EGFP nor the MP^AbMV^_aa1-180_:EGFP mutant altered the MT network in *N. benthamiana* (Figure S3). The microtubules network stayed as intact as in the control cells expressing only the MT marker MAP4:mCherry (Figure S3). Therefore, the question arose, what are the differences determining distinct MP^AbMV^ effects between these two experimental systems?

### 3.7. Examination of the MT Network in Triple Expression Experiments with AbMV MP, Rep, and MAP4:mCherry

**Figure 6:**
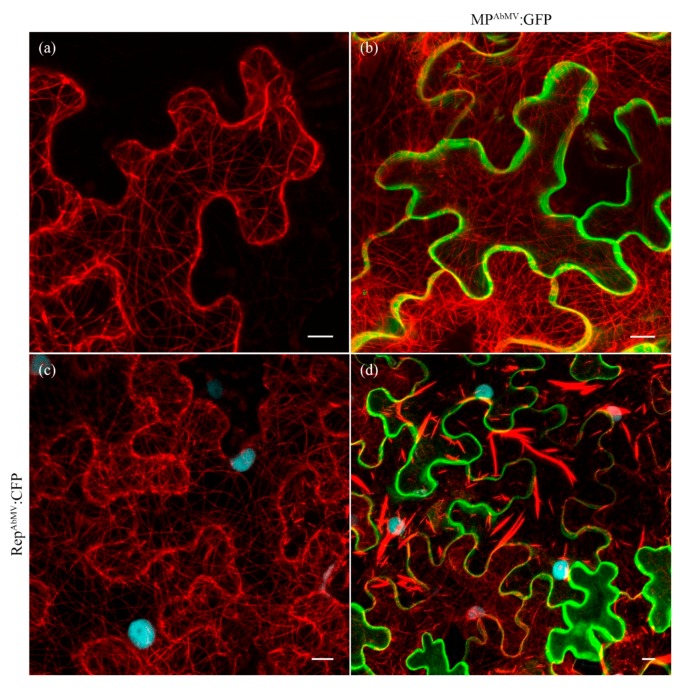
(**a**) Transient expression of MAP4:mCherry; (**b**) co-expression of MAP4:mCherry and MP^AbMV^:EGFP and with (**c**) Rep^AbMV^:CFP; (**d**) triple expression of MAP4:mCherry, MP^AbMV^:EGFP, and with Rep^AbMV^:CFP in epidermal cells of *N. benthamiana* plants. MP^AbMV^ signals in green, MAP4 in red, blue represents Rep^AbMV^:CFP signals. Bar represents 10 µm.

It has been shown that geminivirus infection can activate cell cycle-associated genes and thereby facilitate the transition of infected cells into S phase [5], re-initiating stages preceding cell division. This goes along with changes of the cytoskeletal state. In contrast, the animal cells used in the initial experiments of the study undergo active division cycles continuously. Thus, the replication-associated protein Rep^AbMV^ was included in further experiments to drive the plant cells from G1-phase into S-phase. The MT network was again visualized by expression of the MT marker protein MAP4:mCherry as before, which revealed the typical fishnet-like structure in otherwise non-modified cells (Figure 6a). Co-expression with either MP^AbMV^:EGFP (Figure 6b) or Rep^AbMV^:cyan fluorescent protein (CFP) (Figure 6c) also did not cause any significant alteration of the microtubules network, analogous to the previous tests. However, triple infiltration of all three constructs, MAP4:mCherry, MP^AbMV^:EGFP, and Rep^AbMV^:CFP, induced a conspicuous relocalization of MAP4:mCherry (Figure 6d), resembling the observations in mammalian cells with respect to the MT re-organization; MT appeared as bundled stripes in the epidermal cells. Interestingly, the localization of MP^AbMV^:EGFP (and also Rep^AbMV^:CFP) were unaltered, the MP fusion protein residing mainly at the plasma membrane and the Rep fusion protein within the nucleus.

### 3.8. Identification of Putative Novel MP^AbMV^ Host Interaction Partners

For further functional characterization of factors involved in the obviously indirect MP impact on the MT network of Rep-expressing plant cells, a co-immunoprecipitation/biochemical approach was applied. RFP:MP^AbMV^ and Rep^AbMV^ were both transiently expressed together in *N. benthamiana* leaves and an RFP-trap^®^ enrichment conducted. The co-immunoprecipitation experiment followed by a mass spectrometry analysis identified novel, potential MP^AbMV^ host interaction partners. Mass spectrometry data revealed two potential host factors (Table S3). One interaction candidate was peptidyl-prolyl cis-trans isomerase NIMA-interacting 4 (Pin4), the other one was stomatal cytokinesis defective 2 (SCD2). A control co-immunoprecipitation experiment using free RFP did not pick up both proteins. Both identified MP-interacting candidates were further characterized.

**Figure 7:**
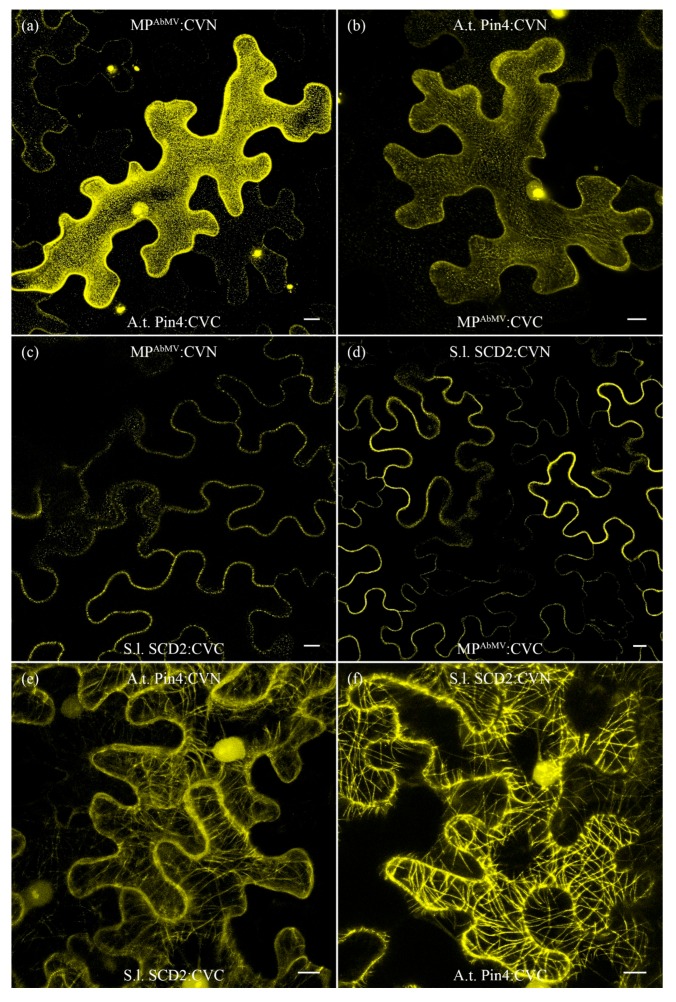
Bimolecular fluorescence complementation of (**a**) MP:YFP^N^ with A.t.Pin4:YFP^C^ (PIN4 = peptidyl-prolyl cis-trans isomerase NIMA-interacting 4); (**b**) A.t.Pin4:YFP^N^ and MP:YFP^C^; (**c**) MP:YFP^N^ + S.l.SCD2:YFP^C^ (SCD2 = stomatal cytokinesis defective protein 2); (**d**) S.l.SCD2:YFP^N^ + MP:YFP^C^; (**e**) A.t.Pin4:YFP^N^ + S.l.SCD2:YFP^C^; and (**f**) S.l.SCD2:YFP^N^ + A.t.Pin4:YFP^C^ in epidermal cells of *N. benthamiana* plants. Bimolecular fluorescence complementation (BiFC) signals in yellow. Bar represents 10 µm.

A.t.Pin4:EGFP localized within the nucleus and at the MT network in transient overexpression experiments in *N. benthamiana* leaves, whereas S.l.SCD2:mCherry localized only to the MT network (Figure S4). BiFC experiments were performed with either MP^AbMV^ and A.t.Pin4, or MP^AbMV^ in combination with S.l.SCD2, in order to confirm in planta complex formation and to identify potential cellular sites of interactions between the viral and the plant proteins. Re-constitution of yellow fluorescent protein (YFP) signals were observed at the cell periphery and in the nucleus for the BiFC combination of MP^AbMV^ and A.t.Pin4, whereas BiFC signals for MP^AbMV^ with S.l.SCD2 were only found at the cell periphery (Figure 7). BiFC analysis also revealed a close association between A.t.Pin4 and S.l.SCD2 (Figure 7). In contrast to the combinations with MP^AbMV^, YFP signals here labelled filamentous structures analogous to those found for expression of A.t.Pin4:EGFP and S.l.SCD2:mCherry (Figure S4). Thus, the BiFC signals most likely colocalized with MTs and support the assumption that MP^AbMV^ redirected A.t.Pin4 and S.l.SCD2 from MTs to the cell periphery. As previously found for the BiFC combination of A.t.Pin4 and MP^AbMV^, YFP signals appeared for the test proteins A.t.Pin4 and S.l.SCD2 within the nucleus, also suggesting that presence of A.t.Pin4 relocalized S.l.SDC2 similar to MP^AbMV^ to the nuclei.

In a follow-up approach, four MP^AbMV^ deletion mutants were tested in BiFC assays with A.t.Pin4, namely pSK03, 04, 05, and 06 (Figure 5a). All BiFC combinations resulted in an YFP signal arising within the nucleus, predominantly in the nucleolus (Figure S5).

These results suggest that MP^AbMV^ has the potential to interact with both factors, and, most interestingly, a begomoviral MP was observed to be located within the nucleus for the first time, which raises many questions and must be addressed in the future.

### 3.9. Transient Silencing of Pin4 in N. benthamiana and Its Effect on AbMV

**Figure 8:**
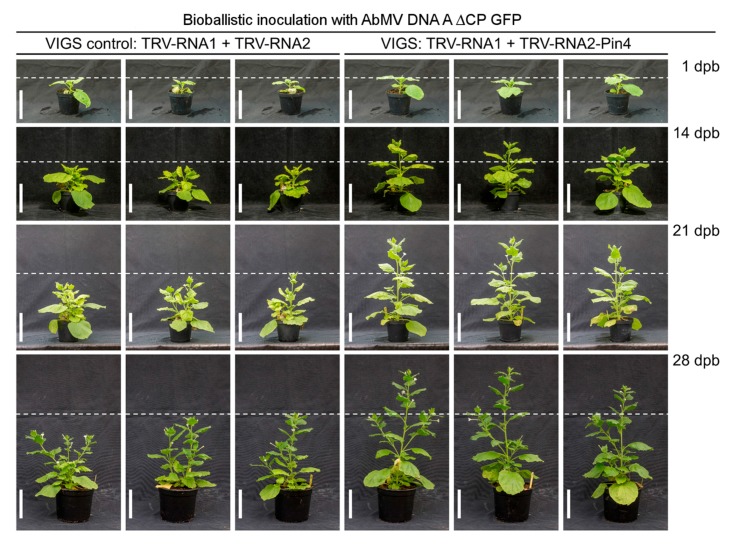
Double inoculation of tobacco rattle virus (TRV) (**left**) or the TRV-Pin4 silencing system (**right**) and AbMV DNA A ∆CP GFP. Bioballistic inoculation was performed 7 days after TRV agro-inoculation. Plants were monitored 1 day post bombardment (dpb), 14 dpb, 21 dpb, and 28 dpb. Plants not silenced for Pin4 are more stunted. Bar represents 20 cm. VIGS = virus-induced gene silencing.

Transgenic *N. benthamiana* plants carrying a dimeric repeat of the AbMV DNA B will release freely replicating DNA B components after inoculation of replication-competent AbMV DNA A delivery constructs [47]. They were applied to provide first indications of the relevance of the putative MP-Pin4 interaction upon systemic begomovirus infections. Young plants were agro-infiltrated with the tobacco rattle virus (TRV) silencing system [48,49] to establish a systemic knock-down of endogenous Pin4, and then bombarded with an infectious clone of AbMV DNA A, where the coat protein was replaced with green fluorescent protein (GFP) [12]. An empty TRV silencing system was agro-infiltrated into control plants. Double-inoculated plants showed a different growth behavior as early as two weeks after the second inoculation step; plants inoculated with the TRV-NbPin4 silencing system grew faster and had a higher amount of DNA A (Figure S6), in comparison to plants inoculated with the empty TRV silencing system, which were also smaller in size (Figure 8). This indicated an effect of Pin4 expression on the progression of a systemic AbMV infection, and may hint at an in vivo relevance of the MP-Pin4 interaction (see discussion below).

## 4. Discussion

The experiments conducted in this study were performed to gain further insight into molecular begomovirus/non-host interactions. As the MP of begomoviruses plays a crucial role in the systemic spread of the virus DNA in plants, the MP^AbMV^ fused to EYFP as well as MP^ClLCrV^:EYFP were expressed in mammalian cell lines. This approach was driven by the hypothesis that analyzing MP in a non-host might lead to new clues for intracellular MP functions, so that the change of the host cell environment might unveil new features of the begomoviral MPs, owing to the distinct biomolecular repertoire of the cell, or as a result of analytical instruments optimized for non-plant cells. After the expression of MP^AbMV^:EYFP or MP^ClLCrV^:EYFP in different mammalian cell lines, both proteins exhibited an unusual curled thread-like cellular localization. The filamentous structures induced by both MPs in mammalian cells led to the initial assumption that cytoskeleton components were involved in their formation. Indeed, a subcellular co-accumulation of MP^AbMV^ and β-tubulin was detected in HeLa cells, thus, the MTs were the most promising candidates of the three cytoskeletal components for co-localizing with MP^AbMV^ or MP^ClLCrV^:EYFP, respectively. Ferralli and colleagues reported in 2006 a dramatic effect of MP^TMV^ on normal MT arrays in mammalian cells [36]. They found hoops of bundled microtubules encircling the nucleus without converging to a centrosome. MP^TMV^ disrupted the MTOC, as determined by an antibody treatment against γ-tubulin which showed reduced detectability of γ-tubulin after MP^TMV^ expression [36]. The filamentous structures of the two begomoviral MPs, however, rather appeared to follow the normal MT stretch directions emerging from the nuclear vicinity, accompanied by a less pronounced but significant bundling. IF were found to co-localize with MP^AbMV^ in HeLa cells as well. In humans, IF proteins are encoded by at least 65 genes, giving rise to a large protein family [50]. They consist of two distinct systems: (i) the nuclear system assembled from lamins constitutes the nuclear lamina [51]; and (ii) the cytoplasmic system, with the widely distributed vimentin, stabilizes the shape of cells [52]. IF are believed to be absent in plants, since they use different mechanisms to stabilize their cells [53]. However, IF antigens have been found along and between microtubules in plants, some form small filamentous structures that still associate with MT [54] while other IF antigens are present but do not form filaments in planta [55]. Plant virus interactions with IF antigens have not been reported before. The co-localization of MP^AbMV^ with IF in HeLa cells could be a side-effect of the interaction of MP^AbMV^ with MT, as the microtubules’ network is also connected to the intermediate filament network.

Structural microtubule-associated proteins (MAPs) participate in the organization of microtubules through binding to the outer MT surface and thereby promoting their stabilization [56,57]. In order to determine whether MP^AbMV^ also could function as an MT stabilization protein, MP^AbMV^-expressing HeLa cells were treated with nocodazole. Under the conditions used here, nocodazole is a microtubule-depolymerizing agent within HeLa cells, interfering with MT stabilization and causing disruption of the MT network. Only two small dots were labelled by immunostaining for MTs, which most likely contained the MTOCs. MP^TMV^ was shown to have a stabilizing effect by binding directly to MT, making them insensitive to nocodazole treatment [36]. A similar effect could be observed after expression of the MT-binding domain of the MAP4 [58]. Different from the findings for tobamoviral MP, MP^AbMV^ could not maintain the filamentous structures in nocodazole-treated cells in our study, but accumulated with β-tubulin in aggregates. This emphasized the role of MT in the formation of the filamentous structures.

The MP^AbMV^ part responsible for inducing the pronounced filamentous structures in mammalian cells was mapped by deletion mutants fused to EYFP. Fluorescence microscopy analysis revealed that the previously identified three functional segments of MP^AbMV^, i.e., the pilot, anchor, and oligomerization domains alone, were not able to trigger the specific filamentous phenotype in the mammalian system.

The pilot domain has been described to direct the protein in planta to either the cell periphery and/or to the nuclear vicinity together with the anchor domain [46]. The single pilot domain was never tested in planta before. The anchor domain was necessary and sufficient to attach the protein to the cell periphery in leaf cells of the host plant *N. benthamiana* and in scale bulb cells of the non-host plant *Allium cepa* [46]. Zhang and colleagues have provided in silico analyses [46], which reveal a putative secondary structure of an amphiphilic protein helix that may have the ability to insert into one leaflet of a biological membrane, anchoring the MP as a monotopic membrane protein. Equally to the other two MP parts, the C-terminal oligomerization domain was dispensable, also suggesting that the assembly of MP homo-oligomers was not necessary to enable the interaction with MTs in mammalian cells.

The minimal MP^AbMV^ segment capable of generating the thread-like structures in mammalian cells, comparable to the effect of full length MPs, comprises the first 180 amino acid residues (here called “association domain”). Interestingly, a truncation of 25 aa at the N-terminus or of 10 aa at the C-terminus of the “association domain” resulted in a loss of the filament-inducing property in HeLa cells.

For the MP of the related begomovirus SLCV, the double alanine substitution of residues Lys112 and Asp113 was shown to change its accumulation from the cell periphery to the cytoplasm in *N. tabacum* cv. Xanthi protoplasts and in insect Sf9 cells [38]. It was still able to mobilize the NSP from the nucleus to the cell periphery, indicating remaining functionality to a certain degree. With the corresponding mutation, MP^AbMV^ lost its ability to induce the “filamentous phenotype” in HeLa cells, which underlines the importance of these two amino acid residues.

The newly discovered interaction of MP^AbMV^ with the MT network in mammalian cell lines led to consecutive testing for MP^AbMV^ tropisms in plant cells. MP^AbMV^ or the mutant MP^AbMV^_aa1-180_, both fused to EGFP, co-expressed each with the MT marker MAP4:mCherry and the replication-associated Rep caused in planta a change in MT structure, presumably resembling the bundling observed in mammalian cells. Different from the findings in these cells, however, MP did not co-localize with the structurally altered MT components in plant cells, which may indicate an indirect effect exerted, potentially, by MP- and MT-interacting partner proteins.

Consequently, we sought to identify plant-encoded factors associated with MP^AbMV^. The viral protein co-precipitated with Pin4 (peptidyl-prolyl cis-trans isomerase NIMA-interacting 4) and SCD2 (stomatal cytokinesis defective protein 2).

*Arabidopsis* SCD2 localizes to putative sites of endocytosis at the plasma membrane, and it is required for cytokinesis and cell expansion [59]. Lewis and Lazarowitz (2009) showed that *Arabidopsis* synaptotagmin A interacted with viral movement proteins [22] and suggested that MPs reach plasmodesmata for cell-to-cell transport of their cargos via an endocytotic recycling pathway. Taken together, this implies that SCD2 may also be involved in virus cell-to-cell transport, which must be proven in further experiments.

Pin4 has a proposed isomerase activity, but its functional role in plants has not been elucidated yet. Human Pin4 (HsPin4; alternative names: Parvulin-14, Parvulin-17) is involved as a ribosomal RNA processing factor in ribosome biogenesis and binds to tightly bent AT-rich stretches of double-stranded DNA [60]. HsPin4 also promotes MT assembly by its peptidyl-prolyl *cis*/*trans* isomerase activity [61]. Interestingly, HsPin4 co-localized in the nucleolus during interphase, on the spindle apparatus during mitosis [62], and was up-regulated during the S and G2/M phases in synchronized human foreskin fibroblast cells [63]. This is consistent with data obtained from a transcriptomic analysis that showed AtPin4 upregulation during CaLCuV geminivirus infection [64].

Interestingly, Pin4 and the MP^AbMV^-Pin4 interaction-indicating BiFC complexes were detected in plant cell nuclei in our study, which is a novel localization of a begomoviral MP. In addition, Pin4-specific BiFC signals inside nuclei also occurred with all tested MP^AbMV^-deletion mutants. MP^AbMV^ does not harbor an obvious nuclear localization signal (NLS), which suggests that it might be directed to the nucleus by Pin4 itself or by further nucleus-directed host factors, where it then has the ability to interact with Pin4. Recently, it was shown for the mammalian prolyl isomerase Pin1 to promote the Herpesvirus-induced phosphorylation-dependent disassembly of the nuclear lamina required nucleocytoplasmic egress of the viral progeny [65]. This raises the question of whether part of the MP^AbMV^ may indeed be translocated into nuclei during natural AbMV infections to prepare the nuclear export of begomoviral movement complexes through interaction with Pin4.

In fact, an initial experiment revealed the first evidence for a role of Pin4 in the viral life cycle: TRV-mediated Pin4 knockdown influenced the systemic AbMV infection. A second possible role of the interaction of MP^AbMV^ with Pin4 may be to perpetuate the cellular S-phase by preventing progression into mitosis, thereby enabling efficient DNA replication of the virus genome.

## 5. Conclusions

The analysis of two begomoviral MPs in mammalian cells and comparative experiments in plant tissue cells have paved the way to the discovery of novel potential plant interaction factors for MP^AbMV^, namely Pin4 and SCD2. In both types of cells, a pronounced influence of the geminiviral MPs on the MT network was observed, with a re-organization of the MTs into bundles. These MP-dependent effects, however, did molecularly differ between the two experimental systems. A clear indication for direct interactions between MP and MTs were obtained in animal cells, whereas adapter or further effector proteins seem to be involved in the effects exerted inside plant cells. In conclusion, this might indicate an involvement of MT in the begomoviral infection cycle, especially since the potential MP interaction partners Pin4 and SCD2 also are associated with MT. Still, it needs to be considered that these observations are based on over-expression of some viral proteins and may be different in a naturally infected cell. However, analogies with animal viral and other cellular trafficking processes, and the first detection of a begomovirus MP inside nuclei, could argue for roles of Pin4 and/or SCD2 in virus movement. Alternatively, or in distinct stages of the infection cycle, however, MP-MT co-localization may also reflect disposal of the viral proteins in non-suitable or non-host cell types. Thus, the biological relevance of these findings needs further molecular dissection in future investigations.

## Supplementary Materials

The following are available online at www.mdpi.com/1999-4915/9/11/334/s1. Figure S1: Cellular localization of MP^AbMV^ transiently expressed in HeLa cells (20 h). Figure S2: Cellular localization studies of EYFP, MP^AbMV^, MP^PNYDV^, P2^CaMV^ in HeLa, MP^ClLCrV^ HeLa, and COS-7 cells. Figure S3: Transient co-expression of A.t.Pin4:GFP with MAP4:mCherry and co-expression of S.l.SCD2:GFP with MAP4:mCherry in epidermal cells of *N. benthamiana* plants. Figure S4: Transient expression of MAP4:mCherry and co-expression with MP^AbMV^ and MP^AbMV(1–180aa)^ in epidermal cells of *N. benthamiana* plants. Figure S5: Bimolecular fluorescence complementation experiments of MP^AbMV^:YFP^N^ with MP:YFP^C^, MP^AbMV^:YFP^N^, and respective MP deletion mutants with A.t.Pin4:YFP^C^, or the reciprocal experiments A.t.Pin4:YFP^N^ with MP^AbMV^:YFP^C^ and deletion mutants in epidermal cells of *N. benthamiana* plants. Figure S6: Quantitative PCR analysis of the viral DNA content in AbMV-infected *N. benthamiana*. Table S1: List of used oligonucleotides for cloning of mammalian cell culture expression constructs. Table S2: List of used oligonucleotides for cloning of plant expression constructs. Table S3: Proteins interacting with RFP:MP^AbMV^ identified by mass spectrometry.
