# Supplementary material for: Begomoviral Movement Protein Effects in Human and Plant Cells: Towards New Potential Interaction Partners
Source: Viruses. 2017 Nov 9;9(11):334. doi: 10.3390/v9110334 (PMC5707541; doi:10.3390/v9110334)
Supplement: Supplementary file 1 [file viruses-09-00334-s001.pdf]

viruses

MDPI

Figure S1: Cellular localization of MP^{AbMV} transiently expressed in HeLa cells (20 hours). Fluorescence microscopy analysis using a primary MP-specific antiserum (a - c) or anti-Myc antibody (d - o) for in situ immunodetection. Red (Alexa Fluor 555-labelled secondary antibody) and green (Alexa Fluor 488-labelled secondary antibody) signals indicate the subcellular distribution of Myc-tagged or unmodified AbMV MP. Nuclei were DAPI-stained and appear as blue. Superposition images of fluorescence emissions are shown on the left (Merge) and individual DAPI and MP-specific signals on the right (Split). A summary of observed localization types (thready, aggregate close to nuclei and homogenous) is given in the table (p). Note that only Myc:MP^{AbMV} exhibited additional cellular localization types. Scale bars represent 10 μm.

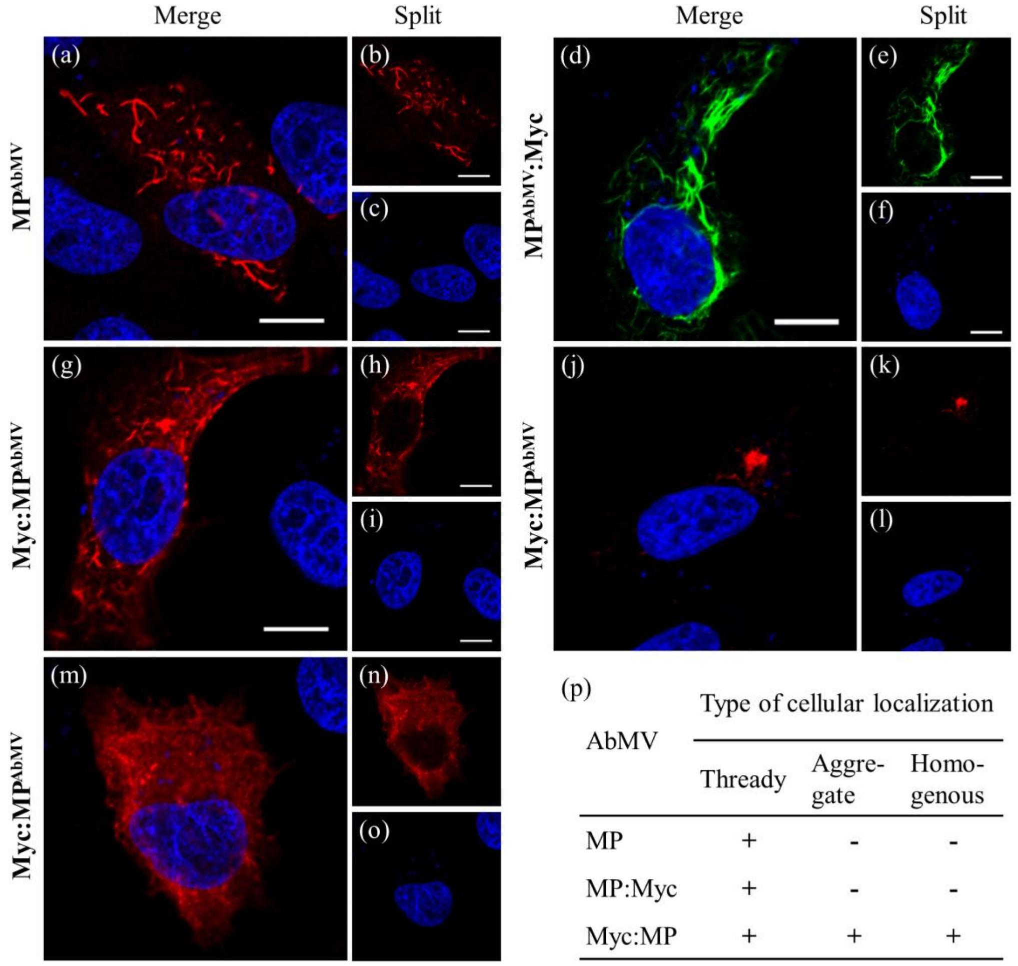

(p)

AbMV	Type of cellular localization		
	Thready	Aggre-gate	Homo-genous
MP	+	-	-
MP:Myc	+	-	-
Myc:MP	+	+	+

Figure S2: Cellular localization studies of EYFP, MP^{AbMV} , MP^{PNYDV} , $P2^{CaMV}$ (a, b, d, e) in HeLa, and MP^{ClLCrV} (c) HeLa and (f) African green monkey kidney cells (COS-7). Blue signal represents DAPI staining. Bar represents 10 μm.

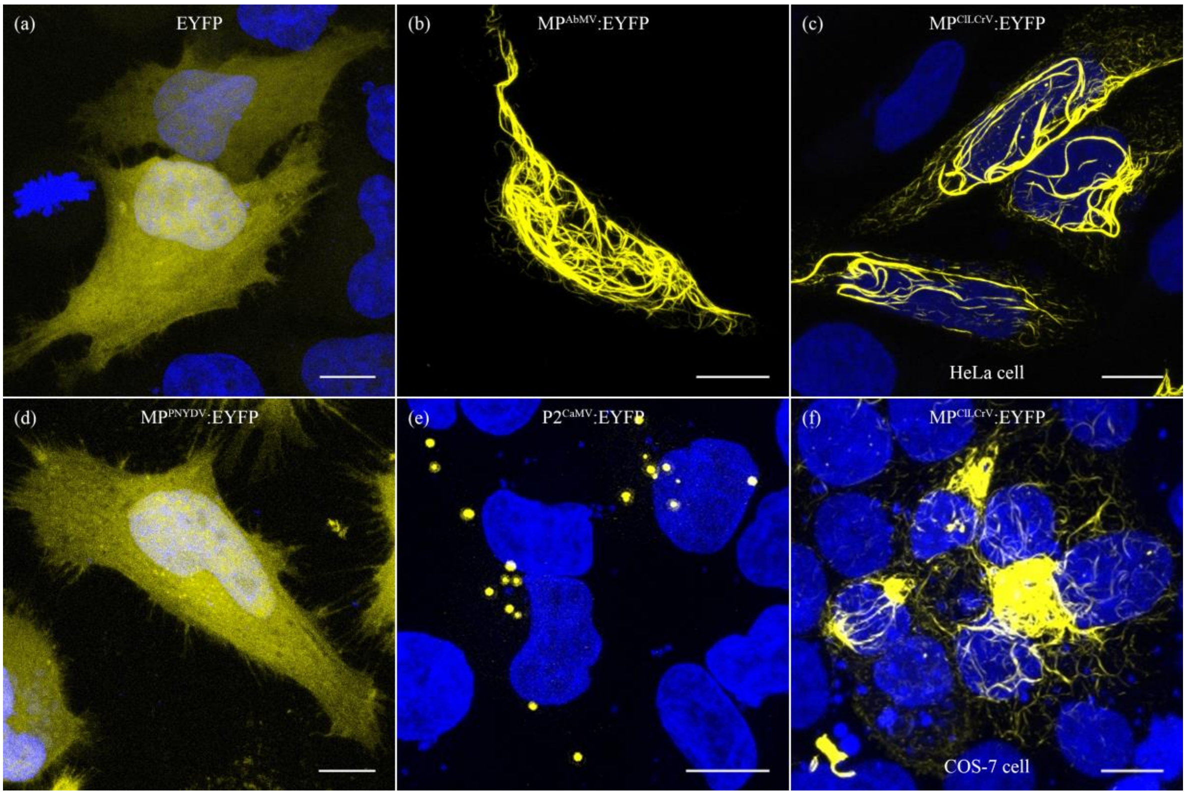

Figure S3: Transient co-expression (a-d) of A.t.Pin4:GFP with MAP4:mcherry, (e-h) co-expression of S.l.SCD2:GFP with MAP4:mcherry in epidermal cells of *N. benthamiana* plants. GFP signals in green, MAP4 in red, co-localization of both signals appears as yellow (b, f; merged pictures). Bar represents 10 μm.

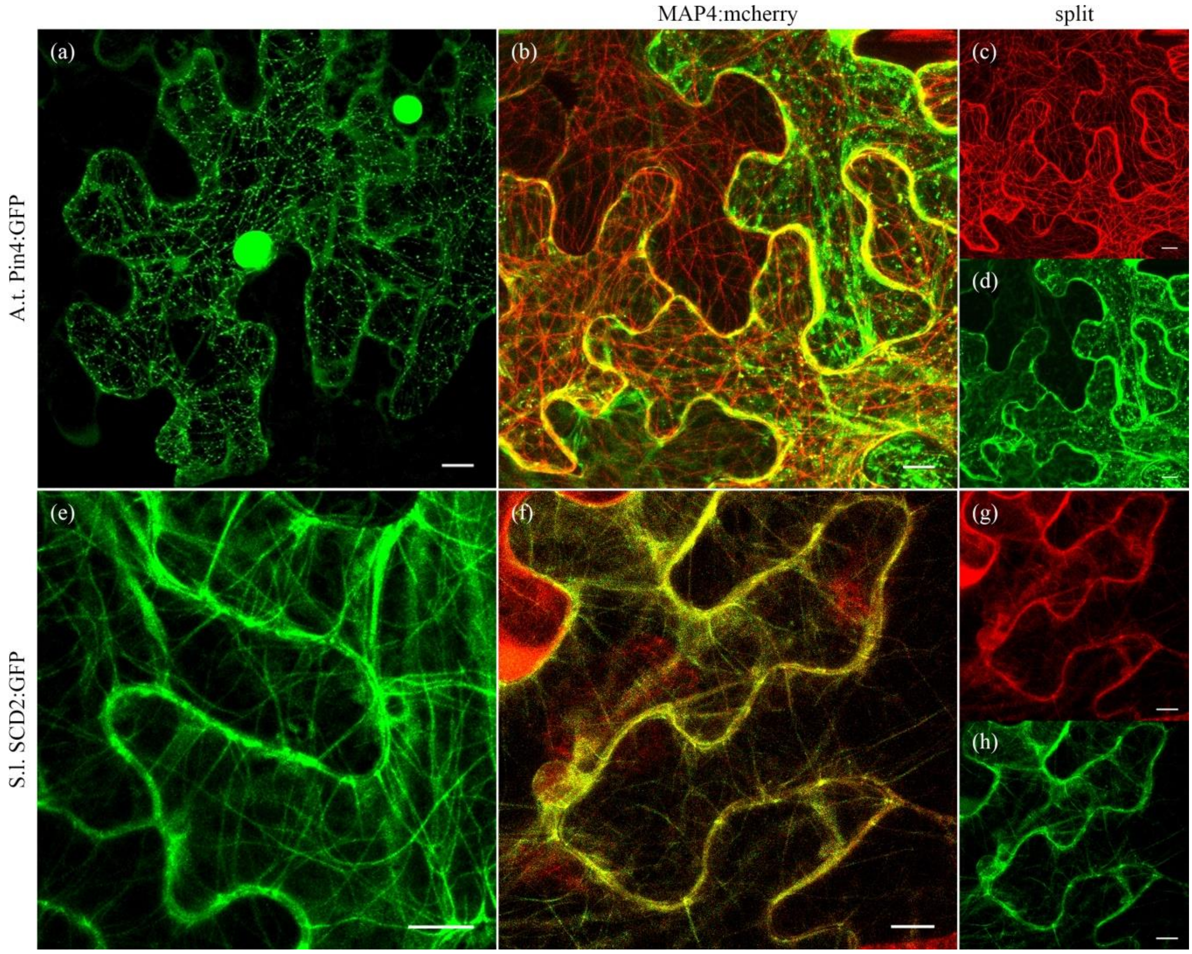

Figure S4: Transient expression (a) of MAP4:mcherry and co-expression with (b) MP^{AbMV} and (c) $MP^{AbMV(1\text{-}180aa)}$ in epidermal cells of *N. benthamiana* plants. GFP signals in green, MAP4 in red, co-localization of both signals appear as yellow in merged pictures. Autofluorescence of chloroplasts are shown in white or blue, respectively. Bar represents 10 μm.

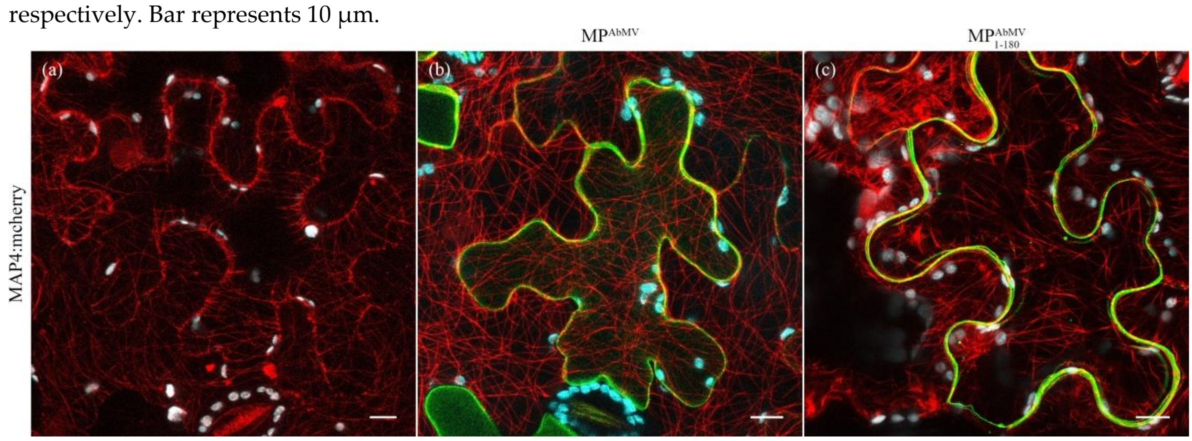

Figure S5: Bimolecular fluorescence complementation experiments of (a) MP^{AbMV}:YFP^{N} with MP:YFP^{C} , (b-e) MP^{AbMV}:YFP^{N} and respective MP deletion mutants (Figure 5a) with A.t.Pin4:YFP^{C}, or the reciprocal experiments (k-o) A.t.Pin4:YFP^{N} with MP^{AbMV}:YFP^{C} and deletion mutants in epidermal cells of *N. benthamiana* plants. BiFC signals in yellow. Pictures (f-j) and (p-t) are merged pictures of (a-e) and (k-o) with brightfield, respectively. Bar represents 10 µm.

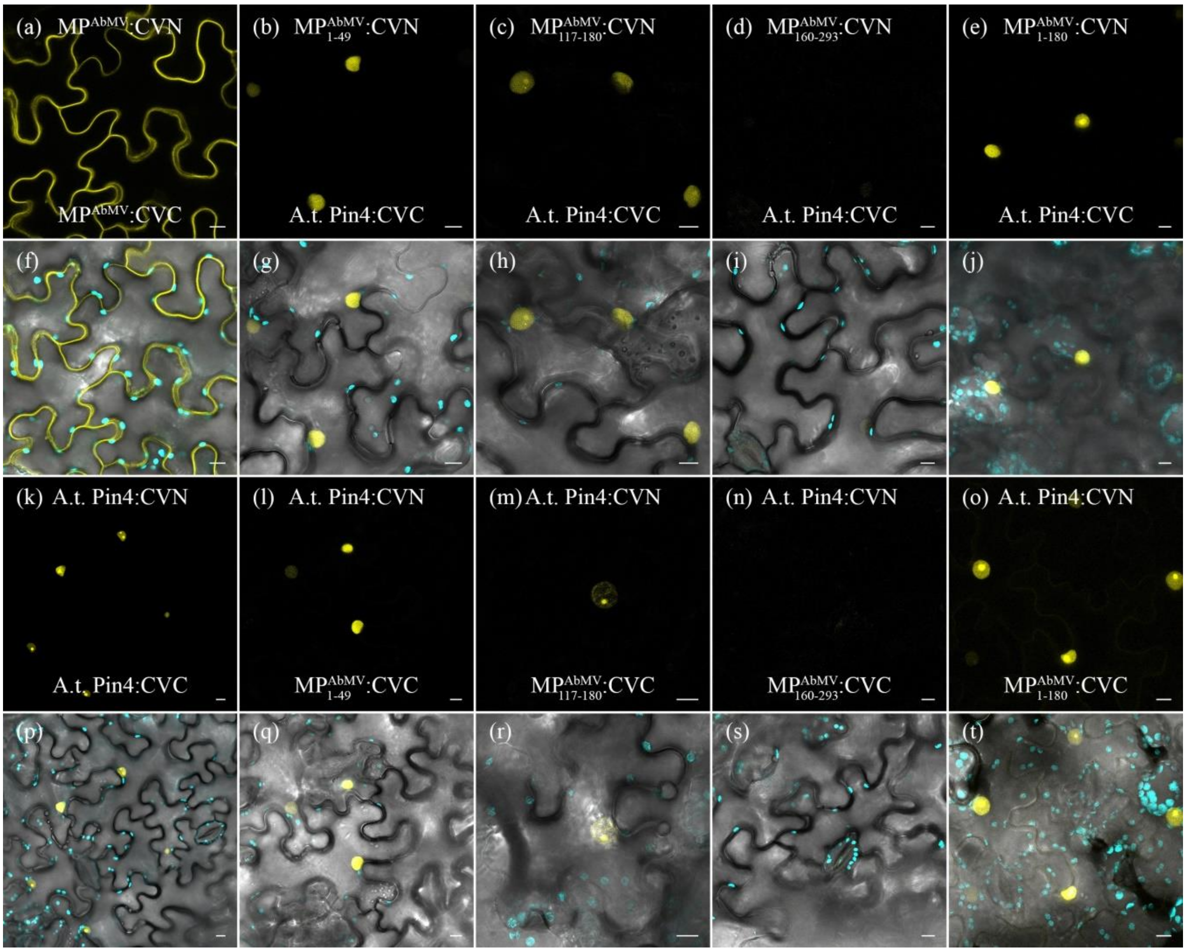

Figure S6: Quantitative PCR (qPCR) analysis of the viral DNA content in AbMV-infected *N. benthamiana*. Plants were either transiently silenced for N.b.Pin4 by a TRV-based system or mock-treated with the empty TRV vectors prior to AbMV application. Relative quantification of AbMV DNA A levels in leaves. Internal reference gene: 25S rRNA gene. Error bars refer to technical replicates.

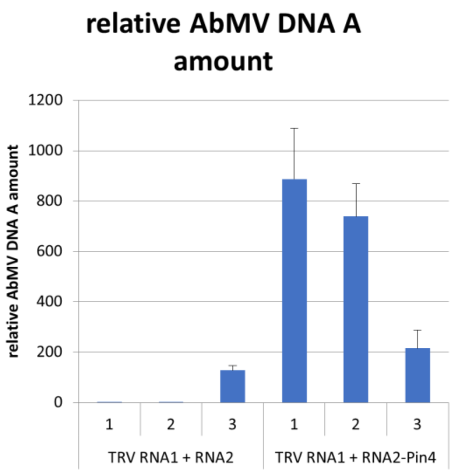

Table S1: List of used oligonucleotides for cloning of mammalian cell culture expression constructs

**Oligonucleotide name**	**Sequence** 5‘ -> 3‘
**MP^{ClLCrV} FP**	AGA ATT CAA TGG AAT CGA AAC TGG TTG TGC C
**MP^{ClLCrV} RP**	TGG ATC CTT TTG TAA AGC CTT GGA TTG CGA AGG G
**MP^{PNYDV} FP**	AGA ATT CAA TGT CGC AAC CTG GAG ATT ATG GAG
**MP^{PNYDV} RP**	TGG ATC CTT AAT CAT TCC TCC TGA CGG AGG
**P2^{CaMV} FP**	GAA TTC ATG AGC ATT ACG GGT CAA CC
**P2^{CaMV} RP**	GGA TCC TTG CCA ATA ATA TTC TTT AAT CCT TCT TTG AT
**MAP4 FP**	AAG CTT ATG TCC CGG CAA GAA GAA GCA AAG
**MAP4 RP**	GAA TTC TGA TCC CGG GCC CAC
**MP^{AbMV}_HA FP**	GAATTC ATG GAT TCT CAG TTA GTA AAT CCT CCG AAC
**MP^{AbMV}_HA RP**	GCGGCCGC TTA AGC ATA ATC TGG AAC ATC GTA TGG ATA TTT CAA TGA TTT GGC
**MP^{AbMV}_1-49 FP**	GAATTC ATG GAT TCT CAG TTA GTA AAT CCT CCG AAC

**MP^{AbMV}_1-49 RP**	GGATCC TT GCT ACG ACT GAG TCT AGC
**MP^{AbMV}_117-180 FP**	GAATTC ATG TGG AAA TTG TAC TAC AAA GTC TGC GAT ACA A
**MP^{AbMV}_117-180 RP**	GGATCC TT TTT CCC ATA ATC CAC ATG GGA
**MP^{AbMV}_160-293 FP**	GAATTC ATG ATA CTG TCC AAA CAG TTC TCC GAA AAA GA
**MP^{AbMV}_160-293 RP**	GGATCC TT TTT CAA TGA TTT GGC TTG AGA AGC
**MP^{AbMV}_1-180 FP**	GAATTC ATG GAT TCT CAG TTA GTA AAT CCT CCG AAC
**MP^{AbMV}_1-180 RP**	GGATCC TT TTT CCC ATA ATC CAC ATG GGA
**MP^{AbMV}_1-150 FP**	GAATTC ATG GAT TCT CAG TTA GTA AAT CCT CCG AAC
**MP^{AbMV}_1-150 RP**	GGATCC TT GAC TGA GTG TTT CGC CG
**MP^{AbMV}_25-180 FP**	GAATTC ATG CAT GAC CTA ACT GAG ATA ATA CTG CAA TTT C
**MP^{AbMV}_25-180 RP**	GGATCC TT TTT CCC ATA ATC CAC ATG GGA
**MP^{AbMV}_K112A/D113A FP**	C TCG CTG GCA GCA CCC ATT C
**MP^{AbMV}_K112A/D113A RP**	G AAT GGG TGC TGC CAG CGA G
**pEYFP-N1/C1 forw**	CCC CCG AAT TCC ATG GAT TCT CAG TTA GTA AAT C
**pEYFP-N1 rev**	CTC TCG GAT CCC CTT TCA ATG ATT TGG CTT GAG

Table S2: List of used oligonucleotides for cloning of plant expression constructs

**Oligonucleotide name**	**Sequence** 5′ -> 3′
**MP^{AbMV} FP**	CACC ATG GAT TCT CAG TTA GTA AAT CCT CCG AAC
**MP^{AbMV} RP**	TTT CAA TGA TTT GGC TTG AGA AGC
**MP^{AbMV}_1-180 FP**	CACC ATG GAT TCT CAG TTA GTA AAT CCT CCG AAC
**MP^{AbMV}_1-180 RP**	TTT CCC ATA ATC CAC ATG GGA
**MAP4 FP**	GAATTC ATG TCC CGG CAA GAA GAA GC
**MAP4 RP**	TCTAGA GAT CCC GGG CCC ACC TCC TG

**Rep^{AbMV} FP**	ACTAGT ATG CCA CCG CCA AAG AAA TTT AG
**Rep^{AbMV} RP**	GGATCC ATG GCC CTC CTC TTG GCC
**MP^{AbMV}:CVN FP**	CACC ATG GAT TCT CAG TTA GTA AAT CCT CCG AAC
**MP^{AbMV}:CVN RP**	TTT CAA TGA TTT GGC TTG AGA AGC
**MP^{AbMV}:CVC FP**	CACC ATG GAT TCT CAG TTA GTA AAT CCT CCG AAC
**MP^{AbMV}:CVC RP**	TTT CAA TGA TTT GGC TTG AGA AGC
**A.t. Pin4:CVN FP**	CACC ATG GGG AAG GAC GCA AAA GCT GG
**A.t. Pin4:CVN RP**	GTT CTT CCT TCC CTC TGA TAA AAT AAT G
**A.t. Pin4:CVC FP**	CACC ATG GGG AAG GAC GCA AAA GCT GG
**A.t. Pin4:CVC RP**	GTT CTT CCT TCC CTC TGA TAA AAT AAT G
**A.t. Pin4:GFP FP**	CACC ATG GGG AAG GAC GCA AAA GCT GG
**A.t. Pin4:GFP RP**	GTT CTT CCT TCC CTC TGA TAA AAT AAT G
**S.l. SCD2:GFP FP**	CACC ATG GAT CGA CGG AGG AC
**S.l. SCD2:GFP RP**	TGG AGA TGC CTC CGA ATC ACT ACT CA
**S.l. SCD2:CVN FP**	CACC ATG GAT CGA CGG AGG AC
**S.l. SCD2:CVN RP**	TGG AGA TGC CTC CGA ATC ACT ACT CA
**S.l. SCD2:CVC FP**	CACC ATG GAT CGA CGG AGG AC
**S.l. SCD2:CVC RP**	TGG AGA TGC CTC CGA ATC ACT ACT CA
**MP^{AbMV}_1-49 FP**	GAATTC ATG GAT TCT CAG TTA GTA AAT CCT CCG AAC
**MP^{AbMV}_1-49 RP**	GGATCC GCT ACG ACT GAG TCT AGC
**MP^{AbMV}_117-180 FP**	GAATTC ATG TGG AAA TTG TAC TAC AAA GTC TGC GAT ACA A
**MP^{AbMV}_117-180 RP**	GGATCC TTT CCC ATA ATC CAC ATG GGA
**MP^{AbMV}_160-293 FP**	GAATTC ATG ATA CTG TCC AAA CAG TTC TCC GAA AAA GA
**MP^{AbMV}_160-293 RP**	GGATCC TTT CAA TGA TTT GGC TTG AGA AGC

**TRV RNA2 N.b. Pin4 FP**	GGATCC ATG GGA AAG GAC TCC AAG GC
**TRV RNA2 N.b. Pin4 RP**	GGATCC TCA ATT TTT CCT CCC TTC AC

Table S3: Proteins interacting with RFP:MP^{AbMV} identified by mass spectrometry.

**Accession** ***(N. benthamiana*)**	**Description**	**Identified peptides**
NbS00041310g0002.1 protein AED:0.34 eAED:0.34 QI:0	AT3G48860.2 (e_value=2e-75) | Symbols: | unknown protein; INVOLVED IN: biological_process unknown; LOCATED IN: plasma membrane; EXPRESSED IN: 22 plant structures; EXPRESSED DURING: 13 growth stages; BEST Arabidopsis thaliana protein match is: unknown protein (TAIR:AT5G23700.1); Has 12429 Blast hits to 9751 proteins in 897 species: Archae - 180; Bacteria - 1190; Metazoa - 6552; Fungi - 1361; Plants - 886; Viruses - 50; Other Eukaryotes - 2210 (source: NCBI BLink). | chr3:18117619-18121853 FORWARD LENGTH=577;; (*ITAG) Solyc03g118390.2.1 (e_value=6e-112) genomic_reference:SL2.40ch03 gene_region:61331348-61340606 transcript_region:SL2.40ch03:61331348..61340606- functional_description:"Chromosome 14 contig 1 DNA sequence (AHRD V1 *-*- Q00WF1_OSTTA)";	NFMDHTPSVR<br>QWSGGSSSTGSSSPAGSPAHPR<br>SGQTPTTHDAVDVER<br>TSAATTTTGAIVPPSR
NbS00018507g0022.1 protein AED:0.42 eAED:0.42 QI:0	gi|217075350|gb|ACJ86035.1| (e_value=2e-69) unknown [Medicago truncatula];; (*SWP) sp|A6QPY8|PIN4_BOVIN (e_value=5e-24) Peptidyl-prolyl cis-trans isomerase NIMA-interacting 4 OS=Bos taurus GN=PIN4 PE=2 SV=1;; (*TAIR) AT1G26550.1 (e_value=3e-70) | Symbols: | FKBP-like peptidyl-prolyl cis-trans isomerase family protein | chr1:9171800-9172716 FORWARD LENGTH=142;; (*ITAG) Solyc12g036200.1.1 (e_value=1e-70) evidence_code:10F1H1E1IEG genomic_reference:SL2.40ch12 gene_region:29804400-29807477 transcript_region:SL2.40ch12:29804400..29807477+ go_terms:GO:0016853 functional_description:"Peptidyl-prolyl cis-trans isomerase (AHRD V1 *-*- Q2F670_BOMMO); contains Interpro domain(s) IPR000297 Peptidyl-prolyl cis-trans isomerase, PpiC-type ";	KGGDLGWFPR
